# MRI of cerebral oedema in ischaemic stroke and its current use in routine clinical practice

**DOI:** 10.1007/s00234-023-03262-2

**Published:** 2023-12-16

**Authors:** Jakub Čivrný, Dorňák Tomáš, Marie Černá

**Affiliations:** 1 Department of Radiology, Palacky University and University Hospital, Olomouc, Czech Republic; 2 Fakultní nemocnice Olomouc, Radiologická klinika, Zdravotníků 248/7, 779 00 Olomouc, Czech Republic; 3 Department of Neurology, Palacky University and University Hospital, Olomouc, Czech Republic

**Keywords:** Ischaemic stroke, DWI-FLAIR mismatch, DWI-ASPECTS

## Abstract

Currently, with the knowledge of the role of collateral circulation in the development of cerebral ischaemia, traditional therapeutic windows are being prolonged, with time not being the only criterion. Instead, a more personalised approach is applied to select additional patients who might benefit from active treatment. This review briefly describes the current knowledge of the pathophysiology of the development of early ischaemic changes, the capabilities of MRI to depict such changes, and the basics of the routinely used imaging techniques broadly available for the assessment of individual phases of cerebral ischaemia, and summarises the possible clinical use of routine MR imaging, including patient selection for active treatment and assessment of the outcome on the basis of imaging.

## Introduction

Cerebral ischemic stroke is one of the leading causes of morbidity and mortality worldwide [1]. Cerebral ischaemia results in an acute functional neurological deficit followed by the development of structural changes—oedema, cell death, gliosis or encephalomalacia [2, 3]. Intracellular swelling followed by interstitial oedema are the first consequences of cerebral ischaemia, and both can be visualised using standard magnetic resonance sequences. Magnetic resonance imaging (MRI) can depict signal alterations within the first hours of the onset of symptoms, starting with signal alteration initially visible on DWI followed by FLAIR [4].

Cerebral ischaemia leads rapidly to a development of irreversible brain damage, also called the ischaemic core, surrounded by tissue that has a functional impairment but is still salvageable—the penumbra [5]. Structural changes start with intracellular swelling also called cytotoxic oedema which may be variably amenable to salvage [6]. The point of no return occurs no later than in the phase of vasogenic oedema, which is distinguished by an irreversible disruption of the blood-brain barrier due to proteolytic damage to basal membrane [7]. The disruption of the blood-brain barrier may be triggered earlier as the breakdown of the blood-brain barrier is preceded by a complicated signalling pathways [8]. Estimation of the reversibility of brain damage is problematic, and yet the differentiation of the core and penumbra is crucial in tailored treatment strategies and in clinical practice is often estimated by means of brain perfusion studies or diffusion-weighted imaging (DWI) [9]. The potential benefit of routine MRI sequences to estimate the volume of the infarct core and also the contribution of basic MRI sequences to the assessment of stroke duration will be discussed later in this paper. This review article describes the essence of early MRI findings seen in arterial cerebral ischaemic stroke, namely what the signal changes visible on DWI and FLAIR in ischaemic stroke truly represent, and summarises the potential use of MRI in ischaemic stroke, including DWI-FLAIR mismatch and the DWI scoring system in clinical practice.

## Pathophysiology of cerebral ischaemic oedema

Brain ischaemia results in changes in homeostasis leading to the development of brain oedema and swelling. Brain oedemas evolve in stages, starting with cytotoxic, immediately followed by ionic and later vasogenic oedemas [10].

**Cytotoxic oedema** is a cellular swelling caused by the failure of ATP-dependent ionic transport systems, leading to the accumulation of Na^+^ and water within the intracellular compartment [11]. It affects all CNS cells, especially astrocytes [11, 12]. For historical reasons, the term ‘cytotoxic oedema’ is used as it was studied following toxicant exposure [13]. Cytotoxic oedema does not itself lead to brain swelling as long as the total amount of water within the brain remains unchanged [10]. However, later it contributes to the accumulation of excess water within cerebral tissue along with extracellular oedema [14].

**Ionic oedema** represents brain swelling caused by the influx of excess water to the brain tissue through capillary walls in the presence of an intact blood-brain barrier [15, 16]. It is triggered by the depletion of ions in the extracellular compartment, especially Na^+^, caused by preceding cytotoxic oedema which creates an **osmotic gradient** for these molecules across the intact blood-brain barrier [17]. The flux of Na^+^, followed by the flux of Cl^−^ and water through intact blood-brain barrier, starts right after the development of cytotoxic oedema in order to maintain the electrical and osmotic balance [15, 18]. This phase is marked by cerebral swelling as the total amount of water is increased. The development of ionic oedema requires perfusion as a source of excessive water—either blood perfusion or possibly perfusion by the glymphatic pathway [10, 19]. The effect of blood perfusion also plays a role in reperfusion injury. The glymphatic pathway is a paravascular transportation system for cerebrospinal fluid. It flushes out water from the cerebrospinal fluid through the cerebral tissue and is facilitated by the aquaporine-4 (AQP-4) channels of the astrocyte endfoot layer of the blood-brain barrier [20]. AQP-4 is a passive water transporter increasing permeability of membranes which means that the amount of transported water depends on ion transport provided by other transporters. Therefore, AQP-4 facilitates water fluxes occurring during ionic oedema and potentiating the development of ionic oedema with the blood-brain barrier still intact [10]. Importantly cerebral ischaemia is followed by AQP-4 overexpression [21]. Animal AQP-4-knockout models showed significantly lower levels of the cellular swelling after ischaemic stroke while no benefits were observed in vasogenic oedema [12, 22].

**Vasogenic oedema** is the next step in the swelling of the brain. It is marked by the progressive accumulation of water in the extracellular compartment of the brain due to all osmotically active molecules now including also proteins as a result of the increased permeability of capillaries to large molecules, e.g. albumin and immunoglobulin G. Plasma protein extravasation is driven by both **hydrostatic** and **osmotic gradients** [10]. It represents an irreversible process which results from increased endothelial transcytosis, structural changes of the cytoskeleton of the endothelial cells, and the contraction of pericytes regulating the integrity of the blood-brain barrier [7]. It is reported that knockout of AQP-4 channels worsens vasogenic oedema thus suggesting protective role of glymphatic pathway in vasogenic oedema [10, 12].

**Fig. 1:**
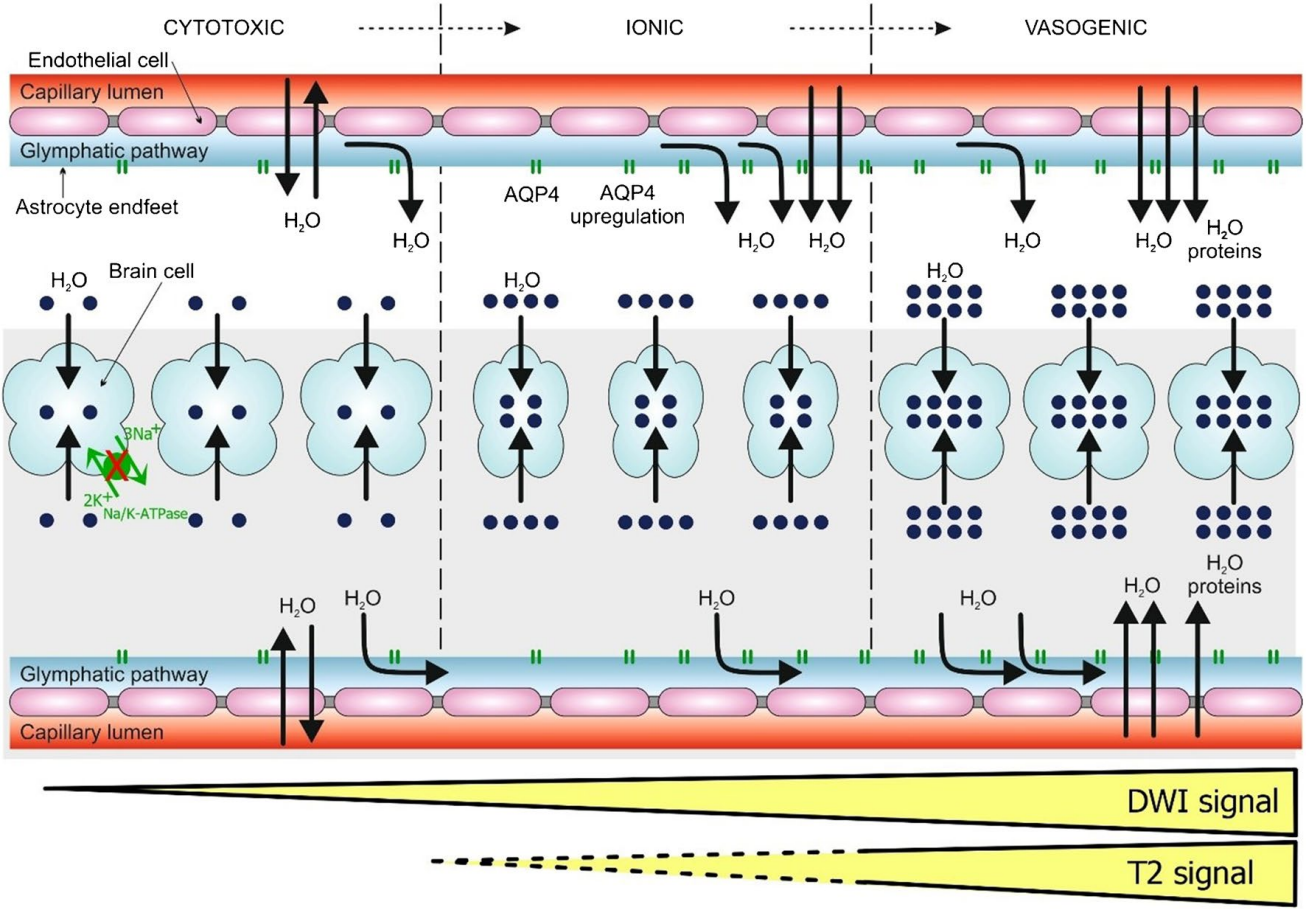
Pathophysiology of phases of cerebral oedema. The image demonstrates the redistribution of water from the extra- to intracellular compartment in cytotoxic oedema and the contribution of blood capillaries and the glymphatic pathway to the development of extracellular oedema. Na/K-ATPase is a transmembrane pump protein which maintains electrochemical gradient. The pump is ATP dependent, and its failure in cerebral ischaemia leads to intracellular swelling. AQP-4 is a passive water channel expressed on the astrocyte endfeet providing clearance of interstitial fluid and solutes via balanced paravascular influx and efflux. AQP-4 is upregulated in cerebral ischaemia. The figure schematically depicts its exacerbating role in the ionic phase and its protective role in the vasogenic phase of the oedema. Note the leakage of the plasma proteins through compromised vascular wall in the phase of the vasogenic oedema. AQP4, Aquaporin-4. Adapted from Reference 10

The shift of water during the early development of oedema is depicted in Fig. 1.

## MRI of cerebral ischaemic oedema

Magnetic resonance imaging can evaluate the physical properties of tissues and liquids by two basic ways, assessing their magnetic relaxation and Brownian motion.

In the phase of **intracellular oedema**, which is defined as an excessive fluid accumulation within cells, the water molecules are locked within the swollen brain cells. Their free diffusion, also known as the Brownian motion, is limited by the cell membranes. The trapped water molecules are thus unable to leave the volume element of tissue under examination (voxel). To assess the diffusivity of the water molecules strong sensitising gradients are applied following excitation. The Larmor frequency (the frequency of precession of the magnetic moment) is dependent on the strength of the external magnetic field. Changing its strength using accessory gradients leads to a change in the precession frequency and consequently a change in the phase of spinning moments of H^+^ of water molecules with unrestricted diffusion. The more distant the motion, the more distinct the change of the magnetic field the water molecules experience. Therefore, variation in the precession frequency of diffusing molecules leads to the loss of phase coherence, which makes the refocusing pulse ineffective. Spins that have lost phase coherence as a result of free diffusion fail to achieve refocusing and do not produce an echo. On the contrary, stationary spins produce an echo after an effective refocusing pulse. The diffusion restriction on diffusion-weighted imaging (DWI) is therefore shown as hyperintense signal. As transverse relaxation also influences the final signal on DWI, an apparent diffusion coefficient (ADC) is calculated, and an ADC map created to assess the true diffusion restriction and eliminate the so-called T2 shine-through effect. A low signal on ADC marks the true diffusion restriction. This inversion relation of the calculated ADC and the measured DWI signal is derived from the mathematical equation for ADC calculation. Cytotoxic oedema is readily detectable using DWI, although the total amount of water within the affected cerebral tissue may remain unchanged [10].

In the phase of **extracellular oedema**, which is defined as an excessive fluid accumulation within interstitial space, the total amount of water within the affected brain is increased; this can be assessed by means of magnetic relaxation analysis. Magnetic relaxation is a process of returning the net magnetisation value following excitation to its original value induced by a strong magnetic field. T1 weighting is aimed at the analysis of T1 (longitudinal) relaxation, T2 weighting at T2 (transversal) relaxation. T1 and T2 relaxation occur simultaneously, although T2 relaxation is significantly faster. In the imaging of T1 weighting the effect of T2 relaxation is intentionally minimised by the setting of a pulse sequence (short repetition and echo times). Similarly, the effect of T1 relaxation on the final T2-weighted image is undesirable and is minimised by the setting (long repetition and long echo times).

**Fig. 2:**
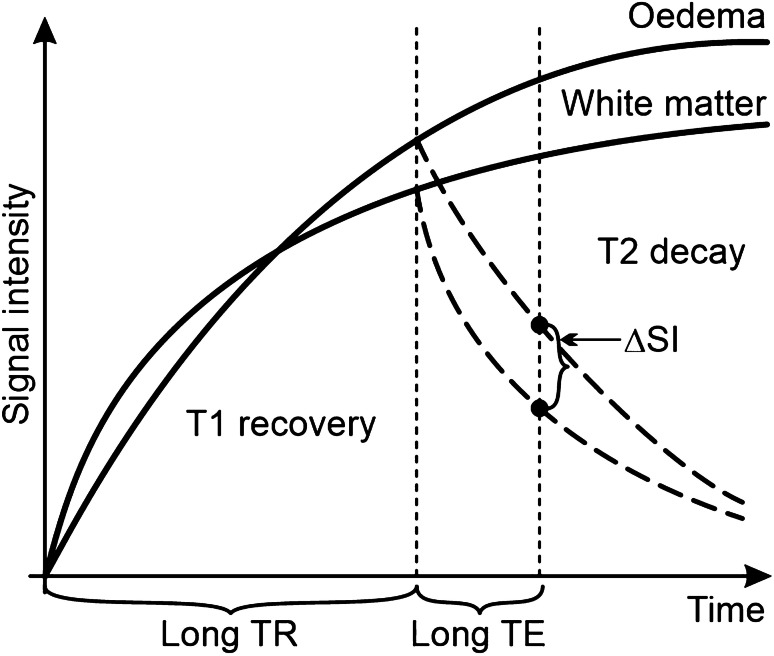
Relation between relaxation rate and signal on T2-weighted pulse sequence. The figure shows the recovery and decay curves of normal white matter and cerebral oedema. Long repetition and echo times depict cerebral oedema as hyperintense. ∆SI, signal intensity difference; TR, repetition time; TE, echo time

The total amount of water influences the final image of the brain. As water molecules have long T1 and T2 relaxation rates, the accumulation of water in oedematous tissue prolongs both T1 and T2 relaxation. While the lengthening of T1 relaxation reduces the T1-weighted signal, the lengthening of T2 relaxation increases the signal. The relation between relaxation and the signal is shown in Fig. 2.

**Fig. 3:**
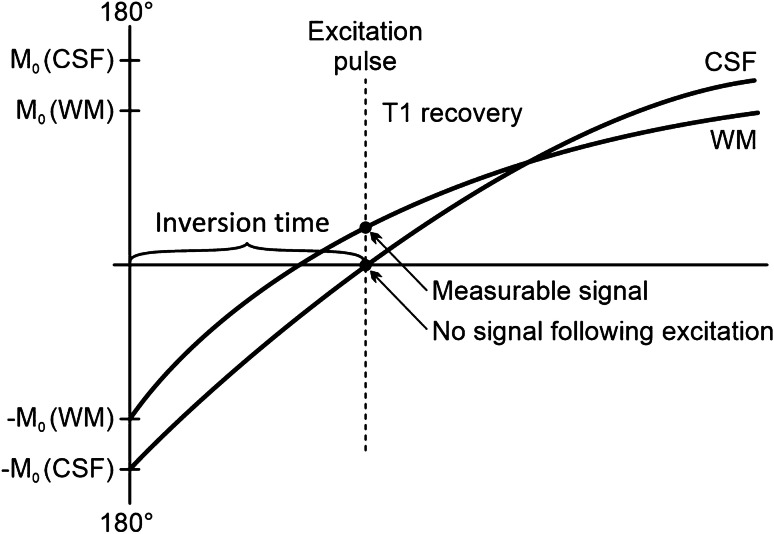
Suppression of the signal of clear fluid on fluid-attenuated inversion recovery. The preparatory inversion pulse flips the longitudinal magnetisation vector through 180°. When the T1 relaxation curve of pure water passes through the null point, the standard excitation pulse is applied. This produces echoes originating in all tissues except pure liquid. Note: the T2 recovery curve is not depicted in the image. M_0_, initial magnetisation vector; CSF, cerebrospinal fluid; WM, white matter; 180°, inversion pulse; TI, inversion time

Because cerebrospinal fluid has a high signal on T2, it makes the visual assessment of hyperintense extracellular oedema difficult. To improve the quality of the visual assessment, the signal of the cerebrospinal fluid is suppressed. Suppression of the signal of the cerebrospinal fluid or any tissue with a known relaxation rate is possible by application of an inversion pulse. The inversion pulse flips the net magnetisation vector through an angle of 180°. The inversion pulse is followed by the standard excitation pulse. The time of the application of the excitation pulse is set to a time point where the tissue to be attenuated has zero net magnetisation. This results in suppression of the signal of the cerebrospinal fluid, being called fluid-attenuated inversion recovery (FLAIR) (Fig. 3). T2-weighted FLAIR is a cornerstone in the evaluation of signal alteration in stroke patients.

It is unclear to what extent ionic oedema can be visualised using T2-weighted imaging. In rats T2 signal changes were best correlated to an increase in brain water accompanied by an increase in protein content rather than brain water or protein content alone [23]. Additionally, no T2 signal change was detected without disruption of the blood-brain barrier, although an increase in the water content of the brain was present.

The signal of water in both a normal and oedematous brain is not suppressed using FLAIR because the macromolecular environment within the brain tissue influences T1 and T2 relaxation. Both the T1 and T2 relaxations of water are shorter in the brain than in pure water [24]. The shortening of T1 relaxation also leads to a change in the inversion time, and therefore the inversion time of FLAIR set to suppress the signal of clear fluid fails to suppress the signal originating from brain tissue.

**Fig. 4:**
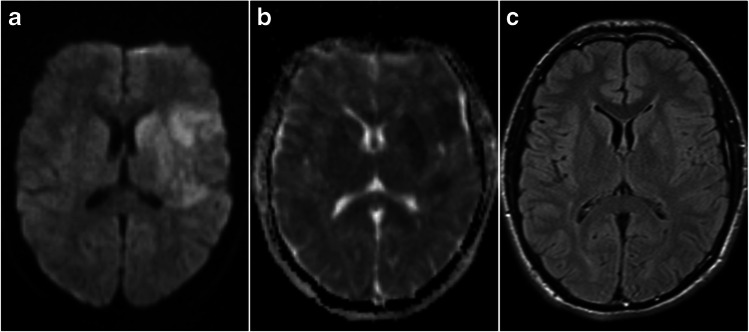
DWI and FLAIR images acquired in the phase of intracellular oedema. **a** The image shows an increased signal on DWI with **b** a corresponding signal drop on ADC and **c** a preserved normal signal on FLAIR

**Fig. 5:**
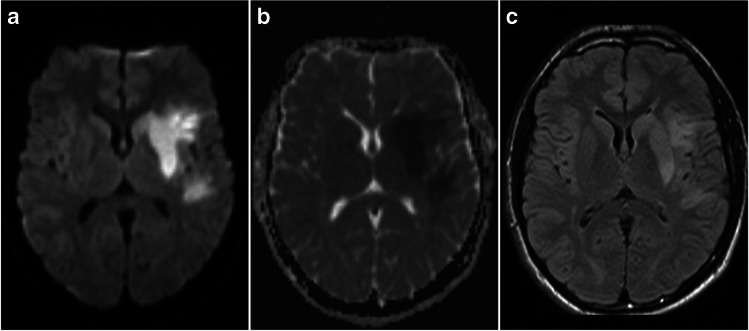
DWI and FLAIR images acquired in the phase of extracellular oedema. Images of the same patient as in Fig. 4, acquired 21 h later. **a** The image shows an increased signal on DWI with **b** a corresponding signal drop on ADC as well as **c** a clearly visible compatible high signal area on FLAIR

The evolution of early signal changes on DWI and T2-weighted FLAIR in ischaemic stroke is depicted in Figs. 4 and 5.

In the later course, as brain cells disintegrate and release water back to the extracellular compartment from the intracellular one, the diffusion restriction starts gradually diminishing. Therefore, the ADC value starts increasing and returning towards normal values (seen as increasing signal on the ADC map). The ADC value typically reaches its normal level in around 1 week’s time in most infarcts and is reported to occur at 2 weeks’ time in brain stem infarcts [25]. However, in the phase of ADC normalisation, the signal intensity seen on DWI remains high. This persisting high DWI signal does not represent true diffusion restriction anymore as it is caused by the so called T2 shine-through effect.

While vasogenic oedema surrounding neoplasms is confined to white matter, signal abnormalities seen in acute ischaemic stroke are present in both grey and white matter [4]. Furthermore, in arterial occlusion ischaemic stroke both intracellular swelling and extracellular oedema co-exist [26].

Cornerstone MR sequences are summarise in Table 1.

**Table 1:** Cornerstone MRI sequences for routine stroke imaging

Sequence	Image	Oedema	Depicts
DWI	Diffusion-weighted	Cytotoxic (intracellular)	Restriction of Brownian motion of water molecules
FLAIR	T2 weighted, CSF signal suppressed	Vasogenic (protein-rich extracellular)	Lengthening of T2 relaxation caused by water molecules

## Application of DWI-FLAIR mismatch in the assessment of the duration of ischaemia

The term ‘DWI-FLAIR mismatch’ describes a lesion which is visible or has a measurable signal change on DWI while continuing to show a normal signal on FLAIR. In other words, it represents an ischaemic lesion in the phase of the intracellular oedema (visible diffusion restriction on DWI) with yet unchanged amount of the total cerebral fluid (normal signal on FLAIR). Therefore, the evaluation of DWI and FLAIR images gives a hint of the duration of the ischaemia. The concept has drawn the attention of neurologists and radiologists in recent years starting in 2010–2011 [27, 28].

The exact evaluation of the duration of ischaemia is complicated by the fact that the infarct growth rate varies among individuals because it is dependent on collateral circulation [29]. Although it is known that the T2-weighted signal correlates with the time from the onset of symptoms [30], the results of studies assessing FLAIR as a predictor of the duration of the stroke within a certain time window are non-uniform [27, 28, 30–34]. However, most of the results suggest that detected DWI-FLAIR mismatch (abnormal finding on DWI and normal finding on FLAIR) can be used as a predictor for a stroke lasting less than 4.5 h. The results are summarised in Table 2.

**Table 2:** Visual evaluation of DWI-FLAIR mismatch in assigning patients to a selected time window

Authors	Number of patients evaluated	Study	Symptoms to MRI	Time group (h)	Sensitivity	Specificity	Support selection based on FLAIR
Aoki et al.	333	Single centre	24 h	0–4.5	74%	85%	Yes
Ebinger et al.	94	Single centre	12 h	0–4.5	46%	79%	No
Cheng et al.	399	PRE-FLAIR study	12 h	0–4.5	58%	78%	-
Petkova et al.	130	Single centre	12 h	0–3.0	94%	97%	Yes
Thomalla et al.	543	PRE-FLAIR study	12 h	0–4.5	62%	78%	Yes
Thomalla et al.	104	Single centre	6 h	0–3.0	48%	93%	Yes
Wouters et al.	206	AXIS-2	9 h	0–4.5	ROC 0.68^a^		-

^a^Correctly classifying 69% of patients with an onset time before or after 4.5 h

A drawback of DWI-FLAIR mismatch is the evaluation of patients who have a subtle signal alteration present on FLAIR. The interrater agreement in such cases is known to be suboptimal or moderate [34–37]. Efforts to increase diagnostic accuracy have been made using quantitative evaluation. This approach is based on measuring signal intensities within the region of interest of the affected and unaffected sides and obtaining the relative signal intensity. The relative signal intensity allows a specific cut-off value to be set. The results are somewhat conflicting. Quantitative evaluation does not outperform visual evaluation universally, but several authors conclude that it can be used in conjunction with visual assessment or in specific scenarios to either improve diagnostic accuracy or support a diagnosis based on visual evaluation [30, 32, 35]. A model that implemented a perfusion study in addition to FLAIR improved the diagnostic accuracy significantly [33]. The results of the studies are summarised in Table 3.

**Table 3:** Quantitative evaluation of DWI-FLAIR mismatch in assigning patients to a selected time window

	Number of patients evaluated	Study	Symptoms to MRI	Time group (h)	Sensitivity	Specificity	RSI threshold	Improvement of visual evaluation
Cheng et al.	399	PRE-FLAIR study	12 h	0–4.5	47%	85%	1.0721	Doubtful
Petkova et al.	130	single centre	12 h	0–3.0	90%	93%	1.0700	Can be helpful
Song et al.	196	LESION Project registry	24 h	0–4.5	78%	78%	1.1500	Suggest use in conjunction with visual evaluation
Wouters et al.	206	AXIS-2	9 h	0–4.5	ROC 0.82^a^		-	In combination with perfusion imaging

^a^Multivariate predictive model integrating quantitative relative FLAIR measurement, information on the severity of hypoperfusion and age correctly classifying 77% of patients with an onset time before or after 4.5 h

*RSI*, relative signal intensity

Visual evaluation of DWI-FLAIR mismatch was used in patients with acute stroke and unknown time of onset to select candidates for intravenous thrombolysis, which resulted in a significantly better functional outcome and more intracranial haemorrhages than a placebo at 90 days [38]. This evidence has been implemented in the updated version of the AHA/ASA Guidelines for the Early Management of Patients with Acute Ischaemic Stroke and is also included in the European Stroke Organisation (ESO) guidelines on intravenous thrombolysis [39, 40]. This approach, introduced in 2019, changed the traditional contraindication of intravenous thrombolysis—unknown time from onset of symptoms, and is in concordance with the finding that a large proportion of nocturnal ischaemic stroke patients with an unknown time of stroke onset have a DWI-FLAIR mismatch suggesting a recent stroke onset [41]. It is of note that DWI lesions larger than one-third of the territory of the middle cerebral artery still represent a contraindication to intravenous thrombolysis [39].

Quantitative DWI-FLAIR mismatch was also used to select patients with an unwitnessed stroke treatable with intravenous thrombolysis within 4.5 h from the time of the discovery of their symptoms and was considered safe in patients without large vessel occlusion [42].

## Application of DWI in the assessment of infarct core volume

As for mechanical thrombectomy, the AHA/ASA Guidelines also implemented new evidence. In selecting patients eligible for mechanical thrombectomy in the anterior circulation who present within 6 to 24 h from when they were last known to be well, CT perfusion or DWI (with or without MR perfusion) is recommended [39]. The eligibility criteria are based on the DAWN and DEFUSE 3 trials [43, 44]. In the DAWN trial the age-stratified mismatch between the severity of the clinical deficit and infarct volume was used to select eligible patients. The infarct volume was assessed with the use of CT perfusion or DWI. Similarly, in DEFFUSE 3 an ischaemic core volume limit was implemented and a mismatch between the infarct core and penumbra was set. Estimation of the volumes of the infarct core and penumbra was based on CT perfusion or DWI and MR perfusion. Both trials used automated image postprocessing software.

However, according to animal models the infarct core estimation based on DWI is imprecise as it includes both reversibly and irreversibly damaged tissue which carries risk of overestimation of infarct core and underestimation of penumbra [45].

## Application of DWI-based outcome prediction

It is a well-known phenomenon that a large volume of a DWI lesion is a predictor of a poor outcome [18, 46]. A DWI lesion volume >80 ml on MRI acquired within 6 h of stroke onset and that of >145 ml when imaged 14 h from stroke onset have been shown to predict rapid early neurological deterioration and the need for neurosurgery owing to the development of malignant oedema [18].

**Fig. 6:**
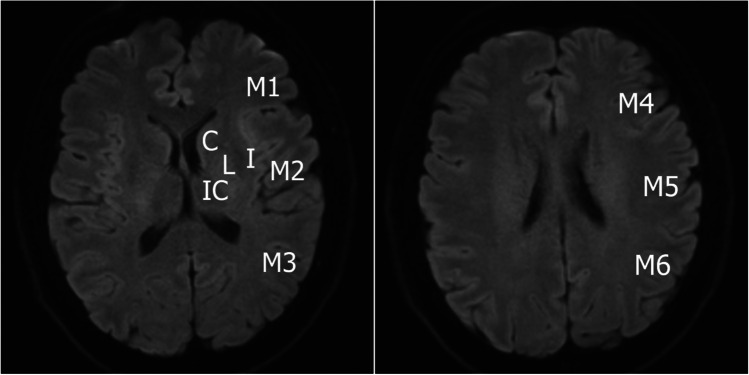
DWI image depicting ten defined regions of the middle cerebral artery territory. One point is subtracted for particular region affected by early ischaemic changes

Analogously to the well-established ASPECTS (Alberta Stroke Program Early Computed Tomography Score), a ten-point CT scoring system evaluating the severity of a stroke with ten points representing normal findings, a similar concept using DWI irrespective of FLAIR has been proposed [47] (Table 4 and Fig. 6). The original CT scoring system was used to select patients with a worse functional outcome at 3 months with the proposed cut-off of ≤7 on a ten-point scale [48]. Currently, the use of ASPECTS is recommended in the AHA/ASA Guidelines to select patients eligible for mechanical thrombectomy in the 0–6-h time window with a score ≥6, although it may be reasonable to include patients with a score <6 as candidates for mechanical thrombectomy as well [39]. However, according to the guidelines further research is still needed. Ongoing studies are supposed to provide evidence on endovascular therapy of large infarct core strokes [49]. A recently published results of SELECT-2 trial suggest that endovascular treatment of patients with low ASPECT improved functional outcome compared to a standard medical care although it was associated with vascular complications [50]. Improving Low ASPECTS Stroke Thrombectomy (I-LAST) study reported benefit of the use of adjusted ASPECTS by means of measurement of water uptake in ischaemic tissue utilising quantitative net water uptake method [51].

**Table 4:** Alberta stroke program early CT score

Normal middle cerebral artery territory	10 points
Affected cortical regions	
M1	−1
M2	−1
M3	−1
M4	−1
M5	−1
M6	−1
Insula (I)	−1
Affected deep grey matter regions	
Lentiform nucleus (L)	−1
Head of caudate nucleus (C)	−1
Involvement of the internal capsule (IC)	−1
Overall score	0–10

Currently for the assessment of ASPECTS both CT and MR are feasible according to the guidelines [39]. The differences between CT and MRI scoring are reported to be small, with CT being preferred because of faster acquisition [47]. The agreement between clinicians evaluating DWI-FLAIR mismatch is reported to be low but increases to substantial when dichotomisation of classification is implemented [37]. Furthermore, the cooperation of a radiologist and neurologist may yield reliable results [52].

It is reported that post-treatment DWI-ASPECTS may be an eligible surrogate of infarction volume for predicting the clinical outcome of stroke patients following endovascular thrombectomy with a score ≥6 predicting good outcomes [53]. DWI-ASPECTS obtained 3 hours after the onset of stroke was predictive of the outcome at 3 months in patients who received intravenous thrombolysis. Those with post-treatment DWI-ASPECTS >6 had good outcomes, with a sensitivity of 88% but low specificity of 33% [54]. Post-treatment DWI ASPECTS as well as successful recanalization and low post-treatment NIHSS were predictors of good clinical outcome. Similarly, patients treated with intravenous thrombolysis within 3 hours were reported to have poor outcomes with baseline DWI-ASPECTS ≤5 and it is suggested that these patients should be excluded from thrombolytic studies before and beyond the 3-h time window [55]. This is not included in the current AHA/ASA Guidelines [39].

It is known that DWI is more sensitive than non-enhanced CT, which means that DWI-ASPECTS might score lower than the traditional ASPECTS. It is estimated that DWI-ASPECTS scores approximately one point lower [56]. Despite these differences, both DWI-ASPECTS and traditional ASPECTS are appropriate tools for the assessment of the prognosis of ischaemic stroke patients [57].

## Reversibility of cytotoxic oedema in acute stroke

On the basis of experimental stroke models, restricted diffusion with a normal T2 signal characterises compromised tissue that may or may not recover with reperfusion [42]. It remains unclear to what extent the volume of cytotoxic oedema seen on DWI represents an irreversible infarct core. Based on animal models no ADC thresholds exist to predict a tissue fate [6]. Cytotoxic oedema of brain cells is potentially reversible if perfusion is restored rapidly [12]. The degree of reversibility of cytotoxic oedema in acute ischaemic stroke has been studied by a number of researchers. Observed rates of DWI lesion reversal between 0 and 83% have been reported [45].

Due to variable reversibility of cytotoxic oedema in acute ischaemic stroke DWI does not precisely depict an infarct core but is used as an approximation.

The infarct core has the potential to grow over time and reach the boundaries of the former penumbra. It has been demonstrated that patients with a significant neurological deficit and low DWI lesion volume have a high probability of infarct growth and early neurological deterioration [58]. The final infarct volume is influenced by the recanalisation status [59], although patients with a DWI lesion with a large volume have poor outcomes despite successful reperfusion [46].

The clinical significance of the reversal of a DWI lesion is unclear. The mean volume of tissue reversal is reported to be small and it is more likely that clinical improvement is related to reperfusion of the collateral penumbra rather than to DWI reversal [46]. A recently published paper contradicted this claim to some extent as it reported that the partial reversal of DWI changes observed in a significant proportion of patients with DWI-ASPECTS ≤5 who were treated with mechanical thrombectomy was identified as a predictor of early neurological improvement and improvement of 3-month clinical outcomes in cases of successful recanalisation [60].

## Current status of MRI in routine practice

For most of the uncomplicated stroke patients CT and CTA is sufficient modality as it is more available and faster than MRI. However, MRI can be more advantageous in certain situations, e.g. in case of clinical uncertainty. Reported rates of stroke under-diagnosis range between 2 and 26% and rates of over-diagnosis from 30 to 43% [61]. Diagnosis may be particularly complicated in case of posterior circulation strokes when clinical symptoms are more diverse and can be misleading. Therefore, in posterior circulation strokes the time to the final diagnosis may be longer compared to anterior circulation strokes [62]. However, negative DWI imaging does not exclude diagnosis of ischaemic stroke. MRI may be beneficial in unknown time from the onset of symptoms and unavailability of perfusion imaging when the approach based on the EXTEND trial is not possible and is based on the WAKE-UP trial (DWI-FLAIR mismatch) [38, 63].

## Conclusion

Intracellular oedema develops within the first hours or even minutes after stroke onset and can be visualised using DWI. It is followed by the development of interstitial oedema which is characterised by brain swelling due to fluid accumulation in cerebral tissue and increased signal intensity on T2-weighted imaging.

DWI may be used for the estimation of infarct core volume in the selection of patients eligible for mechanical thrombectomy who present later than in the traditional time window and for the selection of candidates for intravenous thrombolysis if the time from the onset of the symptoms is unknown. It may be used for the assessment of outcome prediction as the large volume of a DWI lesion is a predictor of poor outcome. The reversal of DWI changes may predict the improvement of the neurological deficit. The degree of reversibility of intracellular oedema is highly variable.

Despite the development of new possibilities for the imaging of patients with acute ischaemic stroke, standard MR imaging keeps its place in diagnostic algorithms.
